# Diagnostic utility of deep tendon reflex responses in rectus femoris and triceps brachii in fibromyalgia: a clinical and electrophysiological study

**DOI:** 10.1007/s00296-025-05808-4

**Published:** 2025-02-21

**Authors:** Ilke Coskun Benlidayi, Volkan Deniz, Ceren Ornek, Aylin Sariyildiz

**Affiliations:** 1 Department of Physical Medicine and Rehabilitation, Faculty of Medicine, Cukurova University, Adana, Türkiye; 2 Department of Physiotherapy and Rehabilitation, Tarsus University Faculty of Health Sciences, Mersin, Türkiye

**Keywords:** Fibromyalgia, Hyperreflexia, Reflex, Knee, Abnormal, Reflex, Triceps, Abnormal, Surface electromyography

## Abstract

The aim of this study was to evaluate deep tendon reflex responses and associated electrophysiological parameters of the muscles in patients with fibromyalgia. This cross-sectional study included 38 patients with fibromyalgia and 32 age- and sex-matched controls. Deep tendon reflexes of the rectus femoris and triceps brachii were tested using a reflex hammer. Electromyographic (amplitude and duration of activation), inertial measurement unit (angular velocity, acceleration), and electromechanical (delay) analyses were performed using a surface electromyography (sEMG) device. Comparative analyses were carried out between patients and controls. Additionally, Receiver Operating Characteristic (ROC) analysis was performed to evaluate the ability of hyperreflexia in distinguishing fibromyalgia patients from controls. Hyperactive deep tendon reflexes in the right/left rectus femoris and/or triceps brachii were observed in more than 85% (ranging from 86.8 to 94.7%) of the fibromyalgia group. Patients with fibromyalgia exhibited significantly increased deep tendon reflex responses compared to controls (*p* < 0.001). Patients revealed significantly higher amplitude, longer duration of muscle activation, greater sagittal acceleration and angular velocity, and shorter electromechanical delay. Normalized muscle activation (right and left rectus femoris and right triceps brachii) in response to deep tendon reflex test showed acceptable ability in differentiating fibromyalgia patients from controls (Area under curve (AUC) = 0.890, 0.784 and 0.782, respectively). For the right rectus femoris, values ≤ 28.3 (clinically corresponding to normoactive/hypoactive deep tendon reflexes) appeared to rule-out fibromyalgia with 94.1% sensitivity and 61.3% specificity. Patients with fibromyalgia exhibit increased deep tendon reflex responses. Normal or decreased deep tendon reflex responses may probably be used as a rule-out criterion for fibromyalgia.

**Supplementary Information:**

The online version contains supplementary material available at 10.1007/s00296-025-05808-4.

## Introduction

Fibromyalgia is a rheumatic disorder characterized by generalized pain in the body, often accompanied by sleep disturbances, mood changes, fatigue, and cognitive defects. The etiopathogenesis of fibromyalgia is complex; it includes genetic predisposition, hormonal imbalances, and various other factors [1]. Recently, the potential role of inflammation in the development of fibromyalgia has attracted considerable attention [2]. This condition is characterized by disruptions in peripheral and central neural mechanisms, mediated by several factors. The functional connectivity pattern in patients with fibromyalgia increases function in certain parts of the brain, yet the exact cause of this abnormal neural communication pattern remains unclear. Specifically, proton magnetic resonance spectroscopy studies have provided some evidence indicating that the anterior insula has increased levels of some excitatory neurotransmitters relative to inhibitory neurotransmitters [3].

In terms of neuromuscular control, there may be differences between patients with fibromyalgia and healthy controls. For instance, the median frequency of differential activations detected by high-density surface electromyogram (sEMG) placed on the trapezius muscle was significantly lower in patients with fibromyalgia than that in healthy controls. The average duration of differential activations was also considerably longer in fibromyalgia patients compared to controls [4].

Neurological issues in fibromyalgia have attracted much attention of researchers. Small fiber neuropathy may occur in 50% of fibromyalgia patients and cause chronic widespread pain [5]. Small fiber neuropathy is now a recognized part of fibromyalgia. Yet, little attention has been paid to any findings of large fiber neuropathy and large fiber neuropathy. The results derived from a large fibromyalgia cohort revealed that electrodiagnostic features of polyneuropathy, muscle denervation, and chronic inflammatory demyelinating polyneuropathy are common in fibromyalgia [6]. Taken as a whole, electrodiagnostic abnormalities can be observed in patients with fibromyalgia [7]. Particularly, the recognition of small fiber neuropathy in patients with fibromyalgia has reinforced the dysautonomia-neuropathic hypothesis. These findings could renovate fibromyalgia concept, diagnosis, and treatment [8].

The aforementioned information raises an important question: Could deep tendon reflexes be altered in fibromyalgia? Changes in deep tendon reflexes may provide insights into numerous central and peripheral neurological disorders. For example, an increase in deep tendon reflexes may be an early sign of corticospinal tract pathologies. Due to decreased supraspinal inhibition, excitability increases and reflex muscle activity is observed in the neighboring motor neuron pools [9]. There is a lack of an understanding regarding deep tendon reflexes and muscle activity in fibromyalgia. Evaluating these parameters would be valuable in enhancing our understanding of the potential effects of fibromyalgia on neuromuscular functions.

We formulated a hypothesis by adhering to the recommendations on scientific hypotheses [10]. The hypothesis of the current study was that: “Deep tendon reflexes are increased, and associated muscle activations are altered in patients with fibromyalgia compared to the normal population.” To test this hypothesis, the objective of the study was to assess deep tendon reflexes and electromyographic parameters of associated muscles in patients with fibromyalgia.

## Patients and methods

### Study design

This case-control, cross-sectional study was conducted at the outpatient clinics of a university hospital. Participants included patients aged between 18 and 64 years who were diagnosed with fibromyalgia according to the American College of Rheumatology 2016 Revisions to the 2010/2011 Fibromyalgia Diagnostic Criteria [11], as well as healthy controls matched by age and gender. The exclusion criteria were: (1) Peripheral nerve lesions, central nervous system lesions/diseases, or other factors (e.g., hyperthyroidism, severe electrolyte imbalance) that could interfere with deep tendon reflexes; (2) Pregnancy; (3) Clinical conditions that prevent proper evaluation of deep tendon reflexes (e.g., severe contracture in the adjacent joint); (4) Clinical conditions in which sEMG examination is not appropriate/permitted (e.g., cardiac stimulants, metallic implants, uncontrolled epilepsy, deep brain stimulators, open wounds). Ethical approval was obtained prior to the study (date: 8-December-2023, number: 139 − 33) from the Local Ethics Committee of Cukurova University. Written informed consent was obtained from each participant.

### Sample size

Sample size was computed by the computer software G*Power 3.0.18 system (Heinrich-Heine-Universität Düsseldorf, Germany) with the selected parameter being: “t-test: difference between two independent means”. The sample size, according to the article by Tsuji et al. [12], having the sEMG measurement as the primary way of evaluation with an allocation ratio of 0.83 and an effect size of 0.8, was calculated to be 38 in the patient group and 32 in the control group. This calculation was made with 90% power and an alpha level of 0.05, resulting in a total sample size of 70.

### Demographic and clinical evaluations

The demographic data of the participants (age, gender, body mass index (BMI), smoking/alcohol use) were recorded. In the patient group, disease duration and details of pharmacological and non-pharmacological treatments were documented. The Revised Fibromyalgia Impact Questionnaire (FIQR) was used to evaluate functionality and disease impact [13, 14]. The Beck Depression Inventory (BDI) and Beck Anxiety Inventory (BAI) were administered to assess psychological status. These are 21-item questionnaires, which each item is scored from 0 to 3. The possible minimum and maximum scores range from 0 to 63. Higher scores represent more severe depressive mood/anxiety [15–17].

Deep tendon reflexes were tested in both the patient and control groups using a reflex hammer (Queen Square™ Hammer, handcrafted reflex hammer redesigned by MDF^®^, MDF Instruments Medifriend Inc., Shangai, China). In the upper extremity, the triceps reflex was recorded, while in the lower extremity, the quadriceps reflex was tested. Reflex testing was conducted with the relevant extremities in a relaxed position. The responses were rated on the following scale: 0 (no response), 1+ (hypoactive), 2+ (normal), 3+ (hyperactive without clonus), and 4+ (hyperactive with clonus) [18, 19]. Reflex testing was conducted three times for each tendon and taps were applied to the tendon with an interval of 5 s between each tap. The demographic and clinical evaluations were performed by medical doctors from the Department of Physical Medicine and Rehabilitation.

### Electromyographic acquisition

Surface electromyography (sEMG) was used to assess the activation of the muscle groups associated with tendons (rectus femoris and triceps brachii-long head) bilaterally. This non-invasive method utilized a wireless, mobile measurement system (TRIGNO, Delsys Inc., Natick, MA, USA), with the following specifications: sampling at 2000 Hz bandwidth 20–450 Hz, input impedance < 10 ohms, baseline noise < 750nV root mean square (RMS), effective EMG signal gain 909 V/V ± 5%, full-wave rectified, and smoothed with a second-order Butterworth low-pass filter. This system is validated for the quantitative determination of deep tendon reflexes [12].

Skin preparation (to ensure stable electrode contact and low skin impedance) and electrode placement were performed according to the recommendations of the Surface Electromyography for the Non-Invasive Assessment of Muscles (SENIAM) project [20]. Lightweight (< 15 g) wireless rectangular sensors (37 × 26 × 15 mm) with parallel bar silver contact electrodes were placed over the muscle belly using double-sided adhesive tape. The electrode was positioned at 50% of the line from the anterior superior iliac spine to the superior part of the patella for rectus femoris, and at 50% of the line between the posterior crista of the acromion and the olecranon, two-finger-width medial to the line for triceps brachii [20].

Following skin preparation and electrode placement, maximum voluntary isometric contraction (MVIC) was performed to normalize (comparison of the voltage obtained from the muscle to a reference value) the sEMG signals collected during deep tendon reflex assessments. For the rectus femoris, participants were seated with the hip at 90° and knee at 45° flexion, and the column aligned. Manual resistance was applied to achieve maximum knee extension [21]. For the triceps brachii, the shoulder and elbow were maintained at 90° abduction and flexion, respectively. Manual resistance was applied to achieve maximum forearm extension [22]. Measurements were taken three times, each with a duration of 5 s per muscle, and data normalization was based on the highest value [23]. After MVIC measurements, raw sEMG signals of the deep tendon reflex-induced muscle activity were collected bilaterally. Electromyographic acquisition was conducted by a physiotherapist with a Doctor of Philosophy (PhD) and medical doctors.

#### Electromyographic analysis

Computerized analysis of the sEMG data was performed by EMGworks^®^ software (version: 4.8.0, Delsys Inc., Natick, MA, USA) and included amplitude and temporal analysis.

##### Amplitude analysis

**Fig. 1:**
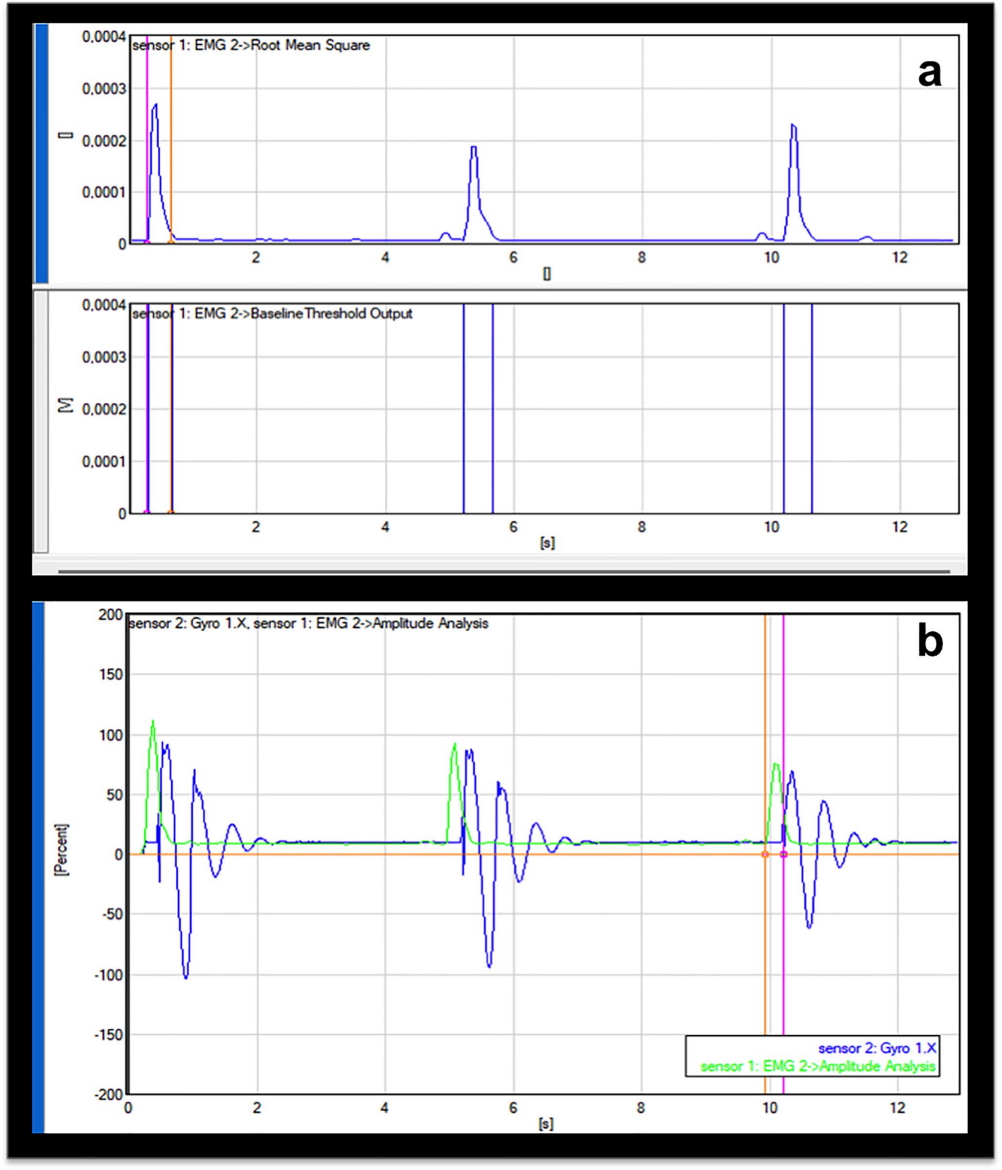
Amplitude of the rectified and smoothed sEMG signal is observed in the upper rectangle and the duration of muscle activation is depicted in the lower rectangle (**a**), the electromechanical delay; the normalized muscle activation is showed in green and the gyroscope signals in blue; the temporal interval between the pink and orange vertical lines indicates the electromechanical delay (**b**)

The values of the rectified sEMG data during deep tendon reflex testing were normalized to the values of the MVIC and expressed as a percentage of the MVIC (%MVIC = muscle activation during deep tendon reflex -RMS/MVIC of the muscle-RMSX100). This process was performed for three deep tendon reflex measurements for each muscle and the average values were recorded (Fig. 1a).

##### Temporal analysis

Duration of the muscle activation is the temporal interval between the onset and offset time of the electrical activity of the muscle. Muscles were considered ‘‘on’’ when their amplitudes were 3 standard deviation (SD) points above the baseline signal for a 25-millisecond window. Muscles were considered to be ‘‘off’’ when the amplitude dropped below 3 SD points above the baseline signal [24] (Fig. 1a).

#### Inertial measurement unit analysis

The inertial measurement unit (IMU) used in this study was integrated into the sEMG sensor (Avanti; Delsys Inc., Natick, MA, USA) by the manufacturer, with temporal synchronization between the IMU and sEMG signals ensured during production. Both IMU and EMG signals were collected simultaneously using a single Bluetooth connection. The IMU recorded triaxial acceleration (± 16 g range) and angular velocity (± 2000 degrees/second range), with a sampling frequency of 2000 Hz and bandwidth 20–450 Hz. To reduce noise and improve signal clarity, a low-pass Butterworth filter with a cut-off frequency of 10 Hz was applied to the signals. The sensors were placed vertically to the midpoint of the leg and midpoint of the forearm and fixed with adhesive tape. The primary kinematic outcomes, including angular velocity (rotational speed) and acceleration of the leg and forearm in the sagittal plane, were derived from the processed IMU data.

#### Electromechanical delay analysis

Electromechanical delay is defined as the latency between the onset of electrical activity in a muscle and the onset of force production by contraction of that muscle. The main reason of electromechanical delay is the latency of force transmission due to the structural slack of the tendon and the non-contractile filaments of the muscle [25]. The value of the electromechanical delay can be used as a useful biomarker for the muscle viscoelasticity such as tone and stiffness [26]. Previous studies revealed that the increased muscle tone makes electromechanical delay shorter than the electromechanical delay of normally functioning muscles [27–29]. In this study, electromechanical delay was measured as the temporal interval between the onset of the sEMG and the onset of the motion production demonstrated by the increasing phase of the gyroscope signals (Fig. 1b).

### Statistical analysis

Statistical analyses were carried out using the Statistical Package for Social Sciences (version 22.0; SPSS, Chicago, IL). Data distribution was assessed using both visual methods (histograms and probability plots) and analytical methods (Kolmogorov-Smirnov test). Categorical data were presented as numbers (n) and percentages (%), parametric data as means and standard deviations, and nonparametric data as medians and interquartile ranges. Comparisons between groups were conducted using the Chi-square test, unpaired t-test, and Mann-Whitney U test. The receiver operating characteristics (ROC) curve was used to determine the sensitivity, specificity, and cut-off values of the rectus femoris and triceps brachii normalized activations (MVCI%) in distinguishing between patients and controls. Area under curve (AUC) values above 0.70 were interpreted as indicating an acceptable diagnostic performance of the test. Alpha value of less than 0.05 demonstrated statistical significance. Correlation analysis was used to examine the potential association of confounders with the dependent variables.

## Results

### Demographics and disease-related data

The study included 38 patients with fibromyalgia (mean age = 44.9 ± 9.4 years) and an age-, sex-matched control group (*n* = 32). The mean BMI, as well as the frequency of smoking and alcohol consumption was also similar between groups (*p* = 0.189, *p* = 0.157, *p* = 0.946, respectively). The patient group demonstrated significantly higher depression and anxiety scores (*p* < 0.001 for both). In the patient group, the median disease duration was 6.0 (7.0) years. The mean FIQR score was 51.6 ± 22.9. Median WPI and SSS scores were 13 (7) and 9 (4), respectively.

### Clinical deep tendon reflex responses

The comparison of clinical deep tendon reflex responses between the patient and control groups is presented in Table 1. In the fibromyalgia group, more than 85% of the participants (ranging from 86.8 to 94.7%) exhibited hyperactive deep tendon reflexes in the right/left rectus femoris and/or triceps brachii. On the other hand, only 2–5 patients displayed normoactive/hypoactive deep tendon reflexes. Compared to the controls, patients with fibromyalgia showed significantly increased triceps and patellar reflex responses on both side (*p* < 0.001 for all).

**Table 1:** Between-group comparison of clinical deep tendon reflex responses

	Fibromyalgia Group (*n* = 38)	Control Group (*n* = 32)	Chi-square value	*p*-value
**Right RF, n (%)**				
No reflex (0) Hypoactive (1) Normoactive (2) Hyperactive (3) Clonus (4)	0 (0.0) 1 (2.6) 2 (5.3) 30 (78.9) 5 (13.2)	0 (0.0) 2 (6.3) 27 (84.4) 3 (9.3) 0	48.820	**< 0.001**
**Left RF, n (%)**				
No reflex (0) Hypoactive (1) Normoactive (2) Hyperactive (3) Clonus (4)	0 (0.0) 0 (0.0) 2 (5.3) 31 (81.5) 5 (13.2)	0 (0.0) 2 (6.3) 27 (84.4) 3 (9.3) 0 (0.0)	51.474	**< 0.001**
**Right TB, n (%)**				
No reflex (0) Hypoactive (1) Normoactive (2) Hyperactive (3) Clonus (4)	0 (0.0) 1 (2.6) 2 (5.3) 30 (78.9) 5 (13.2)	0 (0.0) 2 (6.3) 25 (78.1) 5 (15.6) 0 (0.0)	42.582	**< 0.001**
**Left TB, n (%)**				
No reflex (0) Hypoactive (1) Normoactive (2) Hyperactive (3) Clonus (4)	0 (0.0) 0 (0.0) 5 (13.2) 29 (76.3) 4 (10.5)	0 (0.0) 2 (6.3) 25 (78.1) 5 (15.6) 0 (0.0)	36.025	**< 0.001**

RF, Rectus femoris; TB, Triceps brachii

### Electromyographic, kinematic, and electromechanical deep tendon reflex responses

The comparison of electromyographic, kinematic, and electromechanical deep tendon reflex responses between the patient and control groups is provided in Table 2. For the rectus femoris muscle, the patient group demonstrated significantly higher amplitude, longer duration of muscle activation, higher sagittal acceleration and angular velocity compared to the control group. The electromechanical delay was significantly shorter in the patient group (*p* < 0.001). Regarding the triceps brachii, the amplitude of the muscle activation, sagittal acceleration, and angular velocity were higher, and electromechanical delay was shorter in patients than those in controls. The muscle activation duration did not differ between the groups (Table 2). The distribution of sEMG values according to clinically observed reflex classification is shown in Table 3.

**Table 2:** Between-group comparison of the rectus femoris/triceps brachii activation amplitude and duration, leg/forearm kinematics, and electromechanical delay induced by the deep tendon reflex testing

	Fibromyalgia Group (*n* = 38)	Control Group (*n* = 32)	Z or t value	Effect size (Cohen’s d)	*p*-value
**Rectus femoris**					
**RMS, (MVIC%)** ^**a**^					
Right Left	115.2 (140.2) 119.2 (136.1)	23.5 (24.3) 34.6 (42.7)	Z = -5.671 Z = -4.174	1.844 1.151	**< 0.001** **< 0.001**
**Muscle activation duration, (msec)** ^**a**^					
Right Left	249.5 (47.8) 254.5 (92.5)	208.0 (59.0) 226.0 (89.0)	Z = -4.205 Z = -2.105	1.211 0.567	**< 0.001** **0.035**
**Leg-sagittal acceleration (g)** ^**a**^					
Right Left	0.58 (0.35) 0.55 (0.30)	0.32 (0.14) 0.33 (0.18)	Z = -5.064 Z = -4.086	1.521 1.119	**< 0.001** **< 0.001**
**Leg-sagittal AV, (dps)** ^**ab**^					
Right Left	123.4 ± 50.6 120.8 (80.2)	73.9 ± 42.0 76.8 (63.8)	t = 4.404 Z = -3.148	1.350 0.812	**< 0.001** **0.002**
**EMD, (sec)** ^**b**^					
Right Left	0.18 ± 0.03 0.18 ± 0.03	0.24 ± 0.06 0.24 ± 0.03	t = -5.346 t = -8.035	1.290 1.980	**< 0.001** **< 0.001**
**Triceps brachii**					
**RMS, (MVIC%)** ^**a**^					
Right Left	58.8 (73.4) 52.4 (51.4)	17.9 (26.8) 26.3 (41.8)	Z = -4.268 Z = -2.259	1.186 0.763	**< 0.001** **0.024**
**Muscle activation duration, (msec)** ^**a**^					
Right Left	237.5 (56.7) 238.0 (64.0)	224.0 (100.0) 220.5 (78.5)	Z = -1.538 Z = -0.981	NA NA	0.124 0.327
**Forearm-sagittal acceleration (g)** ^**a**^					
Right Left	0.75 (0.40) 0.71 (0.33)	0.48 (0.43) 0.45 (0.21)	Z = -3.803 Z = -4.289	1.020 1.403	**< 0.001** **< 0.001**
**Forearm-sagittal AV, (dps)** ^**b**^					
Right Left	95.9 ± 30.6 107.5 ± 38.9	67.2 ± 29.9 77.0 ± 34.5	t = 3.944 t = 3.354	0.940 0.820	**< 0.001** **< 0.001**
**EMD, (sec)** ^**a**^					
Right Left	0.18 (0.04) 0.18 (0.04)	0.25 (0.05) 0.24 (0.04)	Z = -6.239 Z = -5.697	2.366 2.075	**< 0.001** **< 0.001**

^a^ Values represent median (IQR), ^b^ Values represent mean ± SD,

RF: rectus femoris, TB: triceps brachii, RMS: root mean square, g: gravity force, AV: angular velocity, dps: degree per second, EMD: electromechanical delay, SD: Standard deviation, IQR: inter-quartile range

**Table 3:** Distribution of normalized muscle activation values ​​according to clinically observed reflex classification

Observed reflex	Right RF-EMG (MVIC%)	Left RF-EMG (MVIC%)	Right TB-EMG (MVIC%)	Left TB-EMG (MVIC%)
Hypoactive Median (25-75%)	*n* = 3* 20.9 (4.6-NA)	*n* = 2* 5.3 (4.4-NA)	*n* = 3* 4.2 (2.0-NA)	*n* = 2* 7.1 (2.7-NA)
Normoactive Median (25-75%)	*n* = 29 23.6 (14.1–50.9)	*n* = 29 45.7 (23.1-101.6)	*n* = 27 21.8 (11.9–48.2)	*n* = 30 26.4 (15.0-60.1)
Hyperactive Median (25-75%)	*n* = 33 95.0 (41.1-174.2)	*n* = 34 91.0 (52.0-192.2)	*n* = 35 54.9 (24.3–92.3)	*n* = 34 51.7 (28.5–72.0)
Clonus Median (25-75%)	*n* = 5 239.6 (190.1-273.6)	*n* = 5 171.6 (142.4-468.2)	*n* = 5 65.0 (30.7–98.6)	*n* = 4 52.5 (25.8-100.2)

RF: rectus femoris, TB: triceps brachii, sEMG: surface electromyography, MVIC: maximum voluntary isometric contraction, NA: not applicable

(*): Since the sample size is less than 4, 75% values ​​could not be given

### Receiver operating characteristic (ROC) analysis

**Fig. 2:**
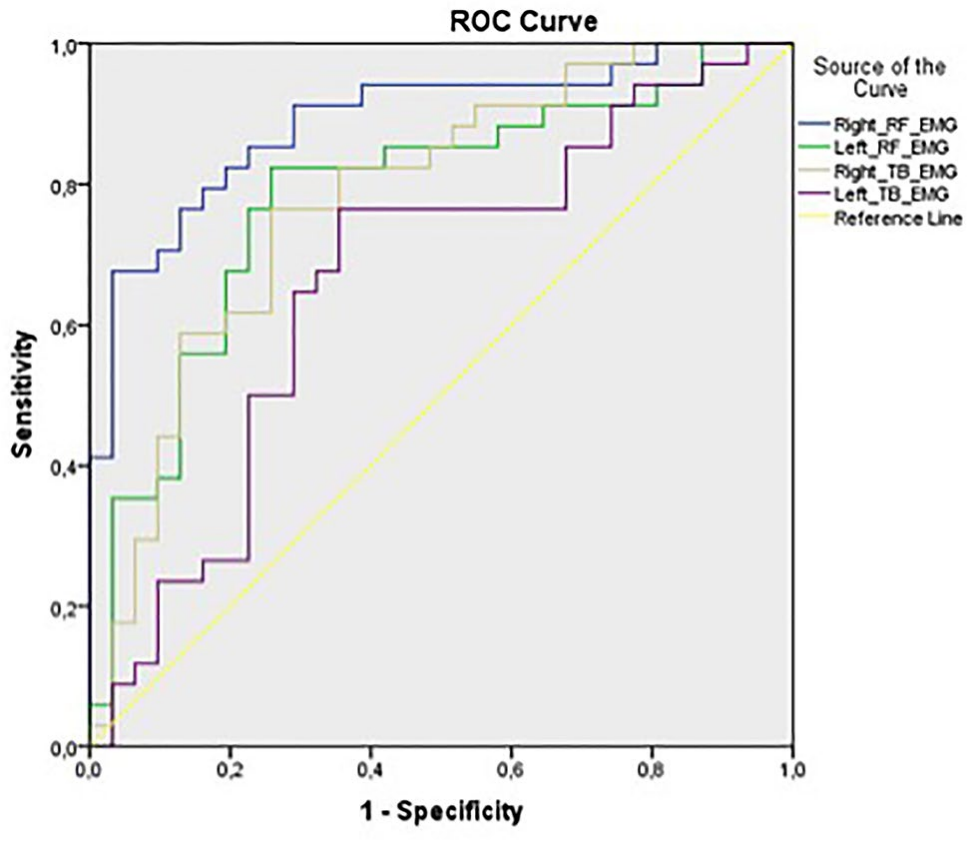
Receiver operating characteristic (ROC) curve of normalized rectus femoris muscle activation values in differentiating fibromyalgia patients from controls

The results of ROC analysis for normalized rectus femoris and triceps brachii muscle activation values during the deep tendon reflex test in differentiating fibromyalgia patients from controls are depicted in Table 4; Fig. 2. Accordingly, the AUC values were above the acceptable level (> 0.70) for the right and left rectus femoris and right triceps brachii (AUC values were 0.890, 0.784 and 0.782, respectively). The sensitivity and specificity of the diagnostic capability varied depending on the cut-off values for muscle activation amplitude.

**Table 4:** Receiver operating characteristic (ROC) analysis of different normalized rectus femoris and triceps brachii muscle activation cut-offs

	Right-RF	Left-RF	Right-TB	Left-TB
**AUC** 95% Cl	0.890 0.810 to 0.970	0.784 0.669 to 0.899	0.782 0.668 to 0.895	0.663 0.527 to 0.799
**Cut-off-1**	> 28.3	> 46.7	> 23.4	> 33.9
Sensitivity (%)	94.1	85.3	82.4	76.5
Specificity (%)	61.3	58.1	64.5	64.5
**Cut-off-2**	> 48.4	> 55.3	> 26.5	> 37.9
Sensitivity (%)	79.4	82.4	76.5	67.6
Specificity (%)	80.6	74.2	74.2	67.7
**Cut-off-3**	> 77.9	> 102.2	> 53.7	> 47.1
Sensitivity (%)	67.6	55.9	55.9	58.8
Specificity (%)	96.8	87.1	87.1	71.0
**p-value**	< 0.001	< 0.001	< 0.001	0.024

AUC: area under the curve, RF: rectus femoris

Patients’ all characteristics, including age, BMI, gender, medication use and smoking, were considered as potential confounders. Correlation analysis revealed that all potential confounders had no significant association with the muscle activation and joint kinematics parameters.

## Discussion

The current study demonstrated that patients with fibromyalgia exhibited increased clinical, electromyographic, and kinematic deep tendon reflex responses compared to individuals without fibromyalgia. Furthermore, muscle activation values in response to deep tendon reflex test showed a reliable capability in distinguishing fibromyalgia patients from controls.

Deep tendon reflex examination is a fundamental aspect of diagnosing neurological diseases. It was first described by Wilhelm Heinrich Erb and Carl Friedrich Otto Westphal in 1875 [30]. This monosynaptic reflex involves a reflex arc composed of two neurons: the afferent neuron and the efferent neuron. The arc includes the receptor organ, afferent neuron, central integrating center, efferent neuron, and effector organ that generates the response. Additionally, this reflex arc is regulated by higher centers, including the corticospinal tract and other descending pathways. The deep tendon reflex is instrumental in assessing neurological disorders that affect afferent nerves, synaptic connections within the spinal cord, motor nerves, and descending motor pathways [31]. Thus, the significant increase in deep tendon reflexes in patients with fibromyalgia may indicate a potential dysfunction or pathology in the afferent nerves, spinal cord, and/or components of the inhibitory pathway. Pathological hyperreflexia holds clinical significance as it indicates a loss of normal inhibitory control over the reflex arc. This heightened reflex response typically emerges when there is damage to an inhibitory structure, most commonly the pyramidal tract [18]. Neuro-hormonal factors and inflammation [1], which result in central sensitization at the medulla spinalis level [32], as well as peripheral sensitization and extended depolarization, may also explain the increased deep tendon reflex responses in fibromyalgia. High prevalence of altered deep tendon reflexes in fibromyalgia patients should be interpreted with caution. There could be confounding factors related to increased deep tendon reflexes in fibromyalgia. On the other hand, further research is required to elucidate the underlying factors related to this enhanced response in patients with fibromyalgia.

Hyperreflexia corresponds to an increase in the range, quickness of response, and expansion in the reflexogenic zone [18]. With high sagittal acceleration, prolonged duration of muscle activation, higher amplitudes and angular velocity, as well as significantly shorter electromechanical delay, we confirmed hyperreflexia electromyographically and electormechanically in patients with fibromyalgia. Clinical deep tendon reflex responses were also significantly increased in the patient group. More than 85% of the patients exhibited hyperreflexia with or without clonus, with this percentage reaching almost 95% in the left rectus femoris. Moreover, only 2–5 patients exhibited normoactive/hypoactive deep tendon reflexes.

To further evaluate the issue, we also examined the potential diagnostic capability of increased deep tendon reflex responses in fibromyalgia. The results revealed that at lower normalized muscle activation (RMS) cut-offs, deep tendon reflex testing had higher sensitivity. However, this was compromised by lower specificity. On the other hand, higher sensitivity corresponds to a higher negative predictive value, meaning that the test performs better as a rule-out test. Conversely, higher cut-offs revealed higher specificity and positive predictive value, making it a better rule-in test [33]. For example, regarding right rectus femoris, a cut-off of 28.3 can be used as a rule-out test with a sensitivity of 94.1% and a specificity of 61.3% (Table 3). Given the high sensitivity at this cut-off, normalized muscle activation values ≤ 28.3, which clinically correspond to normoactive/hypoactive deep tendon reflexes, may be used to rule out fibromyalgia.

Nerve conduction abnormalities can be observed in fibromyalgia [34]. There is some data in the literature related to sEMG findings in patients with fibromyalgia [35–37]. Previous research has demonstrated a higher muscle fibre conduction velocity in muscles of fibromyalgia patients. This is even observed even in non-tender point muscles [36]. Moreover, sEMG studies in fibromyalgia underlined a modified central mechanism related to fibre type recruitment order, which suggested that muscle fatigue is not primarily a muscular problem, rather a complex issue controlled also by the nervous system [37]. Functional abnormalities of the muscle membrane could act as an underlying factor for altered deep tendon reflex responses in patients with fibromyalgia.

Several limitations must be acknowledged. The study primarily involved female patients, which may restrict the generalizability of the findings. However, considering the female predominance of fibromyalgia, this limitation may be less significant in this context. There could be another limitation related to the potential inability to account for all factors that may influence deep tendon reflexes. Normal or decreased deep tendon reflex responses may not necessarily rule-out fibromyalgia, as they could be related to other medical conditions such as radiculopathy in a patient with fibromyalgia. However, we tried to address this point by implementing comprehensive exclusion criteria to ensure a clear and unbiased methodology. There are strengths to be mentioned, as well. The assessment of deep tendon reflex responses was not limited to clinical evaluation; it also incorporated electromyographic, electromechanical, and joint kinematic analyses, providing a more comprehensive assessment. Most importantly, the statistical approach employed in this study enabled us to propose hyperreflexia as a potential diagnostic marker in fibromyalgia.

In conclusion, patients with fibromyalgia exhibit clinical and electrophysiological signs of hyperreflexia. Normal or decreased deep tendon reflex response may probably serve as a rule-out criterion for fibromyalgia. Considering the cases where diagnosis is particularly challenging, implementing this simple assessment method may increase our diagnostic capabilities in daily clinical practice.

## Electronic supplementary material

Below is the link to the electronic supplementary material.

Supplementary Material 1

Supplementary Material 2

Supplementary Material 3

Supplementary Material 4

## Data Availability

The datasets used and/or analyzed during the current study available from the corresponding author on reasonable request.
